# Alcohol and Ganaxolone Suppress Tremor via Extra-Synaptic GABA_A_ Receptors in the Harmaline Model of Essential Tremor

**DOI:** 10.5334/tohm.760

**Published:** 2023-05-18

**Authors:** Adrian Handforth, Hovsep P. Kosoyan, Pournima A. Kadam, Ram P. Singh

**Affiliations:** 1 Neurology Service, Veterans Affairs Greater Los Angeles Healthcare System, Los Angeles, California, United States of America; 2 Research Service, Veterans Affairs Greater Los Angeles Healthcare System, Los Angeles, California, United States of America

**Keywords:** tremor, cerebellum, alcohol, receptor, harmaline

## Abstract

**Background:**

A long-standing question is why essential tremor often responds to non-intoxicating amounts of alcohol. Blood flow imaging and high-density electroencephalography have indicated that alcohol acts on tremor within the cerebellum. As extra-synaptic δ-subunit-containing GABA_A_ receptors are sensitive to low alcohol levels, we wondered whether these receptors mediate alcohol’s anti-tremor effect and, moreover, whether the δ-associated GABA_A_ receptor α6 subunit, found abundantly in the cerebellum, is required.

**Methods:**

We tested the hypotheses that low-dose alcohol will suppress harmaline-induced tremor in wild-type mice, but not in littermates lacking GABA_A_ receptor δ subunits, nor in littermates lacking α6 subunits. As the neurosteroid ganaxolone also activates extra-synaptic GABA_A_ receptors, we similarly assessed this compound. The harmaline mouse model of essential tremor was utilized to generate tremor, measured as a percentage of motion power in the tremor bandwidth (9–16 Hz) divided by background motion power at 0.25–32 Hz.

**Results:**

Ethanol, 0.500 and 0.575 g/kg, and ganaxolone, 7 and 10 mg/kg, doses that do not impair performance in a sensitive psychomotor task, reduced harmaline tremor compared to vehicle-treated controls in wild-type mice but failed to suppress tremor in littermates lacking the δ or the α6 GABA_A_ receptor subunit.

**Discussion:**

As cerebellar granule cells are the predominant brain site intensely expressing GABA_A_ receptors containing both α6 and δ subunits, these findings suggest that this is where alcohol acts to suppress tremor. It is anticipated that medications designed specifically to target α6βδ-containing GABA_A_ receptors may be effective and well-tolerated for treating essential tremor.

**Highlights:**

How does alcohol temporarily ameliorate essential tremor? This study with a mouse model found that two specific kinds of GABA receptor subunits were needed for alcohol to work. As receptors with both these subunits are found mainly in cerebellum, this work suggests this is where alcohol acts to suppress tremor.

## Introduction

Given a prevalence of at least 0.4% [1], 30 million or more persons worldwide have essential tremor (ET). Despite this large number, current treatments are often unsatisfactory, with many patients stopping their medications [2]. To date, drug discovery has been slow and seldom based on molecular targets.

Nineteenth-century clinicians observed that alcohol ameliorated tremor [3]. The sixth U.S. president, John Quincy Adams, who had familial ET, found that without his wine his tremor worsened [4]. Yet how low doses of alcohol reduce tremor remains unexplained. Resolution of this question would not only address an old puzzle but potentially identify a new target for ET therapy.

In controlled settings, alcohol reduces tremor in ET patients from 10 to 90 minutes after oral ingestion, with blood levels of 0.040–0.075 g/dL, below the driving limit in most U.S. states of 0.080 g/dL (17.3 mM) [5,6]. If a small dose is infused into the brachial artery to produce an arm blood level equivalent to that associated with an oral dose that suppresses tremor, the local infusion does not affect tremor, suggesting that alcohol acts within the brain, not in the limb [7]. Two findings implicate the cerebellum in alcohol’s effect. High-density electroencephalography has shown that alcohol-induced tremor amplitude reduction is specifically associated with changes in cerebellar activity [8]. Blood flow imaging with positron emission tomography demonstrated that the cerebellum displays increased activity in ET [9,10,11]. Importantly, an alcohol dose that suppresses tremor, with a blood level of 0.035 g/dL, reduces cerebellar hypermetabolism [9]. This finding suggests that cerebellar cortical neurons are hyperactive and that alcohol reduces this hyperactivity [9]. Insofar as much of the cerebellar cortex is comprised of the massive cerebellar granule cell (CGC) population with its axonal projections, these cells are likely hyperactive in ET, and suppressed by alcohol.

A potential mechanism by which alcohol may reduce cerebellar cortical activity is activation of extra-synaptic GABA_A_ receptors that are located on CGCs. Extra-synaptic GABA_A_ receptors, like synaptic receptors, are composed of two α and two β subunits but incorporate a δ instead of a γ subunit, and exert tonic rather than phasic inhibition. In these receptors, δ is usually associated with α4 subunits throughout the brain, but on CGCs α6 is the associated partner and is intensely expressed here; whereas α4 levels in the cerebellum are much lower, being expressed in the Purkinje cell (PC) layer and molecular layer in mice [12,13]. CGCs from α6 knockout (KO, α6^–/–^) mice lack GABA-mediated tonic inhibition [14]. The location of α6βδ receptors on CGCs, where they respond to GABA released by Golgi neurons, provides a mechanism for controlling the excitatory CGC drive to PCs.

Alcohol in levels as low as 3 mM enhances GABA-mediated tonic currents by recombinant α6βδ and α4βδ GABA_A_ receptors on oocytes [15], and in levels as low as 10 mM in CGCs in slices [16,17]. In addition, the activation of extra-synaptic receptors on CGCs by alcohol leads via an indirect circuit mechanism to increased GABA release by Golgi neurons, so that synaptic GABA_A_ receptors are activated as well [18], thus contributing to inhibition of CGC activity. Alcohol fails to enhance GABA-mediated tonic currents or to enhance GABA release from Golgi neurons in cerebellar slices from δ^–/–^ mice [18].

Based on alcohol effects on α6βδ CGC GABA_A_ receptors at levels below the driving limit, we postulated that alcohol suppresses tremor by activating these receptors, so that δ and α6 subunits are required for alcohol’s anti-tremor action. To test this hypothesis, we utilized the mouse harmaline model, in which tremor is driven by rhythmic, coupled inferior olivary (IO) bursting [19]. Harmaline tremor is a symptom model, in which the brain areas activated during tremor overlap with the tremor circuit revealed by magnetoencephalography in ET [20], including the cerebellum [21], thalamus, motor cerebral cortex and brainstem [19]. This extensive circuitry overlap Is consistent with considerable pharmacologic overlap, in which many drugs exert similar actions on ET and harmaline tremor [22].

As an independent test of the hypothesis that activation of α6βδ GABA_A_ receptors can suppress tremor, we examined the action of the neuroactive steroid ganaxolone on harmaline tremor. Ganaxolone is a derivative of allopregnanolone modified to resist degradation and avoid hormonal effects. Neuroactive steroids potentiate GABA-mediated tonic currents in slices of dentate granule cells (expressing α4βδ) and of CGCs (expressing α6βδ), but not if slices are taken from δ KO (δ^–/–^) mice [23]. Ganaxolone, 10 mg/kg, exerts anxiolytic effects in wild-type (WT, δ^+/+^) mice, but not in δ^–/–^ mice [24]. We therefore sought to determine whether ganaxolone suppresses harmaline tremor in α6- and δ-subunit dependent fashion.

## Methods

### Study design

Our objectives were to demonstrate that low-dose alcohol and ganaxolone can each suppress harmaline tremor in WT mice, and to determine whether littermate mice lacking either the δ or α6 GABA_A_ receptor subunit fail to respond to this action. Because alcohol is rapidly cleared by mice [25], an effect of alcohol on tremor was anticipated to occur only in the first post-injection epoch (E1). In the case of longer acting ganaxolone, all post-injection E1 to E4 epochs were evaluated. Mice were selected, as the harmaline model is well-established in mice, and GABA_A_ receptor subunit-null genotypes are available for this species. Mice were assigned randomly to dosing groups, and the quantitation was performed by automated software. Animal protocols conformed to the National Institute of Health’s Guide for the Care and Use of Laboratory Animals (NIH Publications No. 80–23, revised 1978), and were approved by the Veterans Affairs Greater Los Angeles Institutional Animal Care and Use Committee. All efforts were made to minimize animal suffering and to reduce the number of animals used.

### Animals

δ^–/–^ (δ KO, *Gabrd^–/–^*) mice were donated by the University of California at Los Angeles, where they had been backcrossed for over 11 generations with C57BL6/J mice. α6^–/–^ (α6 KO, *Gabra6^–/–^*) mice were obtained from Jackson Laboratories (Bar Harbor, ME). These had been generated with a 129x1/SvJ x 129S1/Sv cell line inserted into a C57BL6/J blastocyst and were backcrossed with δ^+/+^ mice in our laboratory for 10 generations. Heterozygote mice were interbred to produce offspring that were genotyped with polymerase chain reaction (Transnetyx, Memphis, TN) and δ^+/+^, δ*^–/–^*; and α6*^+/+^*, α6*^–/–^* littermates respectively used for experiments. Both sexes were used as adults, and mice had *ad libitum* access to food and water.

### Test procedures

To ensure that doses of alcohol or ganaxolone used in harmaline experiments did not cause psychomotor impairment that could non-specifically suppress tremor, we first tested these drugs in the straight wire test, a highly sensitive test for drug-induced impairment [26]. The ability of adult δ^+/+^ mice not receiving harmaline to pass the test at various alcohol or ganaxolone doses was assessed. Results were confirmed with α6^+/+^mice. In this test, a mouse is suspended by the front paws from a rigid, 2-mm diameter wire. For the mouse to pass at a specific drug dose, it had to stay on the wire at least 10 seconds and touch the wire with a hind paw within those 10 seconds, and do so on each test conducted at 10-minute intervals for one hour following drug administration. A drug dosage passed only if all 6/6 mice passed all such testing.

Testing at various doses sought to determine the highest dose at which 6/6 mice passed; only this or lower doses were utilized in subsequent harmaline experiments. Each mouse received any drug or harmaline only once.

To assess motion power, each mouse was placed on an 8.1-cm diameter mesh on top of a 24.1-cm high cylinder that rested on a Convuls-1 Replacement Sensing Platform model 1335-1A (Columbus Instruments, Columbus, OH), fitted with a load sensor, connected to a Grass model P511 AC amplifier (Grass Instruments, West Warwick, RI) with 1 and 70 Hz filter settings. Digitally recorded motion power was analyzed using Spike2 software (Cambridge Electronic Design; UK) to perform Fourier transformation of the data into frequency spectra. Data were sampled at 128 Hz. Prior experience indicated that in mice harmaline-induced tremor occurs at 9–16 Hz, creating a corresponding motion power peak on digital frequency spectra [27,28]. To avoid changes in tremor power due merely to changes in overall activity level, this tremor-associated bandwidth motion power was divided by background overall activity motion power to form the measure of analysis, *motion power percentage* (MPP): (9–16 Hz motion power)/(0.25–32 Hz motion power) x 100, as previously described [28].

Mice were acclimated to the platform, then 15 minutes of pre-harmaline baseline motion data collected, then harmaline (Sigma-Aldrich, St. Louis, MO), 20 mg/kg in 4 ml saline/kg injected subcutaneously. Once tremor had developed, within 5 minutes, motion power was again assessed during two successive 15-minute epochs with an intervening 5-minute rest in the home cage. Drugs or vehicle were injected intraperitoneally in a volume of 10 ml/kg at the end of the second harmaline tremor epoch. Ethanol (Thermo Fisher, Canoga Park, CA) was injected in doses of 0, 0.40, 0.50, or 0.575 g/kg in saline. Motion power accession was re-initiated 10 minutes after injection for four more 15-minute epochs on the elevated platform (E1 to E4), with intervening 5-minute rests. Procedures with ganaxolone (Tocris Bio-Techne, Minneapolis, MN) were the same, but the doses 0, 3.5, 7, 10 mg/kg, prepared in 45% (2-hydroxypropyl)-β-cyclodextrin (Sigma-Aldrich) diluted 1:1 in saline, were used.

### Data analyses

Mean MPP values were compared using a repeated measure (mixed) analysis of variance (ANOVA) model. A repeated measures model was utilized as MPP values in each animal were measured repeatedly across time epochs from baseline to E4. Residual errors were examined using normal quantile plots (not shown) to confirm that the errors have a normal distribution, as required by this parametric model. The Shapiro-Wilk test for normality confirmed that the errors followed a normal distribution. The model-based means and pooled standard errors (SEs) were analyzed and p values determined for dose comparisons at each receptor genotype and time. Mean comparisons under the repeated measure ANOVA model were carried out using the Fisher least significant difference (LSD) criterion, after Miller, 1981, section 2.7 [29]. The Fisher LSD allows comparisons among the four dose levels such that the overall chance of a false positive (type I error) is alpha = 0.05 or less. Computations were performed using R 4.0.5 (R Foundation for Statistical Computing, Vienna, Austria, https://www.R-project.org/).

## Results

### Tremor suppression by low-dose alcohol requires GABA_*A*_ receptor δ and α6 subunits

**Figure 1:**
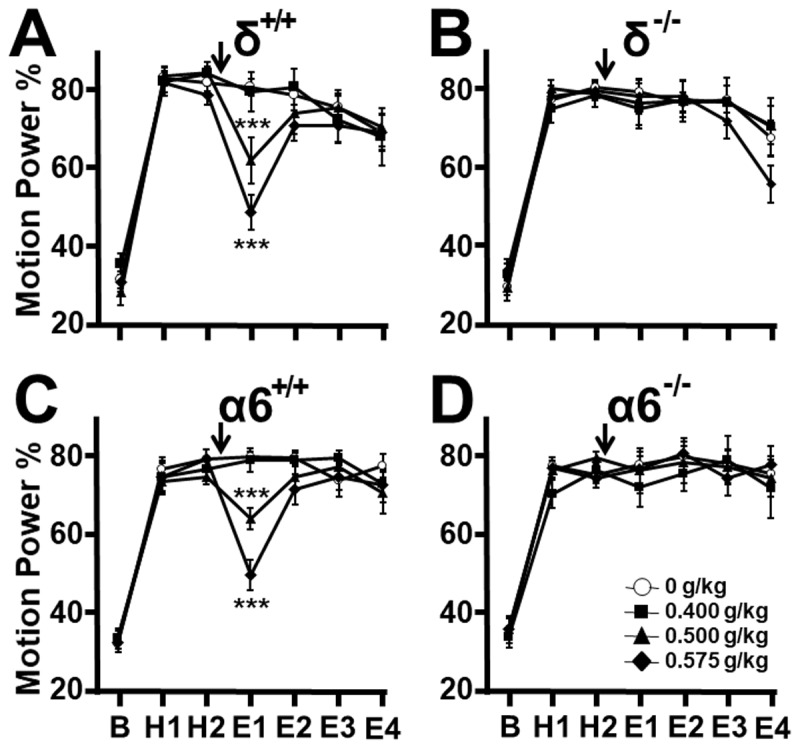
**Alcohol effect on harmaline tremor**. Motion power in groups of mice followed sequentially during 15-minute epochs at baseline (B), pre-treatment harmaline (H1, H2), and after vehicle or alcohol injection (arrow, E1–E4). **(A)** In δ^+/+^ mice ethanol, 0.50 and 0.575 g/kg, suppressed tremor during E1 compared to vehicle controls but **(B)** not in δ ^–/–^ littermates. **(C)** Similarly, in α6^+/+^ mice ethanol, 0.50 and 0.575 g/kg, suppressed tremor during E1 compared to vehicle controls but **(D)** not in α6 ^–/–^ littermates. * P > 0.05, ** P > 0.01, *** P > 0.001, ANOVA.

In δ^+/+^ and α6^+/+^ mice, the motion power percentage (MPP) that fell by chance within the 9–16 Hz bandwidth approximated 30–35% during the 15-minute pre-harmaline baseline (B) (Figure 1A, 1C). With harmaline administration, motion power became dominated by tremor, so that the MPP approximated 75–83% during the two 15-minute harmaline pre-treatment epochs (H1, H2).

The alcohol dose 0.575 g/kg was chosen as the highest tested dose, as 6/6 δ^+/+^ mice passed all straight wire tests at this dose, whereas not all passed at 0.600 g/kg. Based on published pharmacokinetic data in mice [25], 0.500 and 0.575 g/kg are estimated to produce blood levels of 0.06 and 0.07 g/dL respectively at the midpoint of the first post-injection 15-minute epoch (E1), comparable to the blood level of 0.040–0.075 g/dL associated with tremor suppression in ET [5,6,30].

Following injection of saline vehicle or alcohol 0.40, 0.50, or 0.575 g/kg in δ^+/+^ mice, (*n* = 11, 11, 12, 11 respectively), tremor was reduced by the 0.500 and 0.575 g/kg doses during post-treatment epoch E1 compared to the vehicle group (Figure 1A, p < 0.0001, p < 0.0001 respectively), but not at 0.400 g/kg (p = 0.6318). Tremor in the 0.500 and 0.575 g/kg groups recovered to control levels during the following epochs, consistent with rapid alcohol clearance.

Littermate δ ^–/–^ mice exhibit normal behavior and are indistinguishable from δ^+/+^ mice. They displayed pre-harmaline baseline and pre-treatment harmaline MPP values comparable to those of δ^+/+^ mice, indicating no alteration in harmaline tremor response. Figure 1B displays motion power in 11, 11, 12, 11 δ ^–/–^ mice receiving vehicle or alcohol 0.40, 0.50, 0.575 g/kg respectively, and shows that, in contrast to δ^+/+^ mice, 0.500 and 0.575 g/kg failed to reduce tremor during E1 (p = 0.6410, p = 0.9179 respectively). These findings indicate that the extra-synaptic GABA_A_ receptor δ subunit is required for tremor suppression by low-dose alcohol.

As with δ^+/+^ mice, 6/6 α6^+/+^ mice passed the straight wire test at the alcohol dose 0.575 g/kg. After α6^+/+^ mice were injected with vehicle or alcohol 0.40, 0.50, or 0.575 g/kg (*n* = 12, 11, 12. 12 respectively), tremor was reduced by the 0.500 and 0.575 g/kg doses during post-treatment epoch E1 compared to the vehicle group (Figure 1C, p = 0.0009, p < 0.0001 respectively), but not at 0.40 g/kg (p = 0.890).

Littermate α6^–/–^ mice appear normal, without motor anomalies, and displayed normal baseline and pre-treatment MPP harmaline values, indicating a normal tremor response (Figure 1D). On treatment with vehicle or 0.40, 0.50, 0.575 g/kg alcohol (n = 12 all groups), in contrast to littermate WT mice, 0.50 and 0.575 g/kg alcohol failed to reduce tremor during E1 (p = 0.7981, p = 0.9317 respectively). These findings indicate that the GABA_A_ receptor α6 subunit is required for tremor suppression by low-dose alcohol.

### Tremor suppression by ganaxolone requires GABA_*A*_ receptor δ and α6 subunits

**Figure 2:**
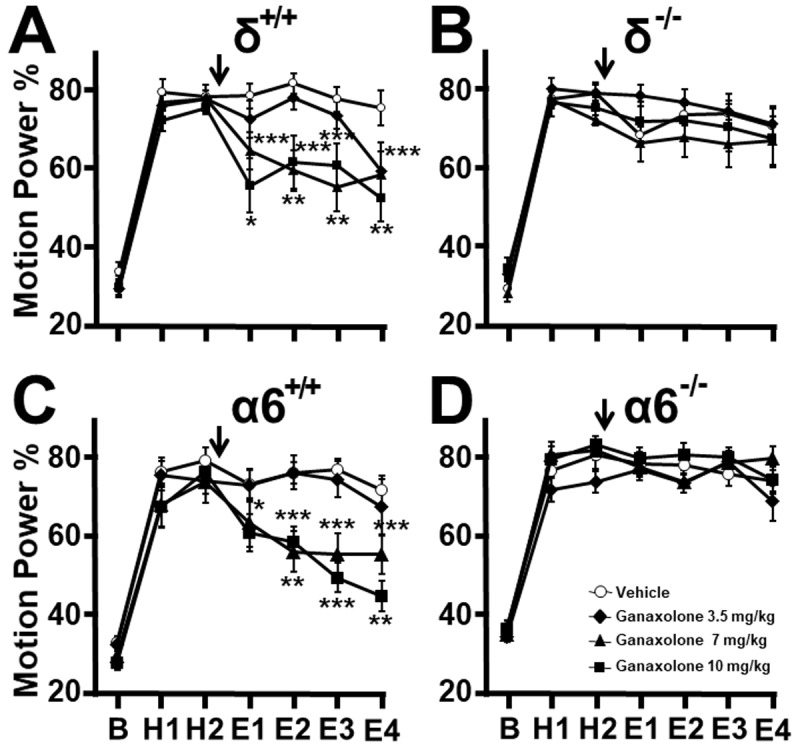
**Ganaxolone effect on harmaline tremor**. Motion power during baseline (B), pre-treatment harmaline (H1, H2), and after vehicle or ganaxolone injection (arrow, E1–E4). **(A)** In δ^+/+^ mice ganaxolone, 7 and 10 mg/kg, suppressed tremor following injection ompared to vehicle controls but **(B)** not in δ ^–/–^ littermates. **(C)** Similarly, in α6^+/+^ mice ganaxolone, 7 and 10 mg/kg, suppressed tremor compared to vehicle controls but **(D)** not in α6 ^–/–^ littermates. * P > 0.05, ** P > 0.01, *** P > 0.001, ANOVA.

In δ^+/+^ mice the highest dose of ganaxolone passed by 6/6 animals in straight wire testing was 10 mg/kg and therefore this was the highest dose used. This dose was lower than the dose 33 mg/kg required to produce ataxia on the rotarod test [31]. On injection of ganaxolone 0, 3.5, 7, and 10 mg/kg to 11, 11, 10, 11 δ^+/+^ mice respectively, 3.5 mg/kg had no significant effect on tremor (Figure 2A) compared to the vehicle-treated group, while 7 mg/kg reduced tremor during E1 to E4, (p = 0.0002, 0.0011, 0.0067, 0.0002 respectively). The dose 10 mg/kg also suppressed tremor during E1 to E4 (p = 0.0164, < 0.0001, < 0.0001, = 0.0030 respectively). In contrast, when δ^–/–^ littermate mice were administered vehicle, 3.5, 7, 10 mg/kg ganaxolone (n = 11 all groups), no tremor suppression occurred in any epoch at any dose (Figure 2B).

As with alcohol, findings with α6^+/+^ mice replicated those seen with δ^+/+^ mice as expected given the extensive backcrossing of our α6 colony with δ^+/+^ to achieve a uniform genetic background. In straight wire testing, 6/6 α6^+/+^ mice passed the test at the highest ganaxolone dose used, 10 mg/kg. In tremor experiments, vehicle or ganaxolone 3.5, 7, 10 mg/kg was injected into α6^+/+^ mice, n = 12 all groups. The dose 3.5 mg/kg exerted no effect on tremor compared to cyclodextrin vehicle-treated controls. The dose 7 mg/kg reduced tremor during post-injection epochs E1 to E4 (Figure 2C, p = 0.0189, 0.0010, < 0.0001, < 0.0001 respectively). The dose 10 mg/kg caused borderline reduction at E1 (p = 0.0618) and reduced tremor during E2 to E4 (p = 0.0002, 0.0001, 0.0021, respectively). In contrast, when vehicle or ganaxolone 3.5, 7, 10 mg/kg was administered to littermate α6^–/–^ mice (n = 12 all groups), no dose exerted tremor suppression in any epoch compared to vehicle controls (Figure 2D). These results indicate that the δ and α6 GABA_A_ receptor subunits are needed for these doses of the neurosteroid to suppress tremor.

## Discussion

CGCs intensely express α6 GABA_A_ receptors, both synaptic, with γ2, and extra-synaptic, with δ subunits. In δ^–/–^ mice, cerebellar α6 is not reduced, but γ2 expression is increased, reflecting a compensatory increase in α6βγ2 receptors [32,33]. A deletion of α6 causes more profound changes, so that 50% of cerebellar GABA_A_ receptors are lost, with both synaptic and extra-synaptic receptors severely affected as indicated by depletion of δ and γ2 subunits [34,35]. Yet α6^–/–^ mice display no motor deficits and are agile [34,35]. Upregulation of voltage-independent potassium conductance in CGCs appears to underlie the compensation [14], and offers an explanation why these KO mice display normal motor function. Moreover, the expression of harmaline tremor depends on the integrity of a circuit involving IO, PCs, and the deep cerebellar nuclei (DCN), discussed below, which lack α6βδ GABA_A_ receptors.

We found that alcohol in low doses estimated to produce blood levels comparable to those associated with tremor reduction in ET suppressed harmaline tremor in WT mice, but not in KO littermates lacking the α6 or the δ GABA_A_ receptor subunit. The failure of the KO mice to show tremor suppression is not due to enhanced alcohol metabolism, as ethanol pharmacokinetics are normal in these mice [36,37].

Outside the cerebellum, α6 is expressed in the trigeminal ganglion [38], cochlear nuclei [39,40], and faintly in the spinal trigeminal nucleus [13]. In the cochlear nuclei, α6 and δ appear not to be expressed in the same cells [39]. In these locations, α6 GABA_A_ receptor activation is unlikely to affect tremor. Within cerebellum, α6 expression is virtually limited to the CGC layer [13], so that this is the likely site of alcohol’s anti-tremor action. Concerning the requirement for the δ subunit for alcohol’s anti-tremor action, it is logically conceivable that alcohol has a two-target effect on both α4βδ GABA_A_ receptors in an unknown location and on α6βγ2 receptors that are abundant on CGCs but, given that α6βδ and α4βδ receptors are comparably sensitive to alcohol [15], and that they are much more sensitive to alcohol than are α6βγ2 GABA_A_ receptors [15], this interpretation appears unlikely. The most plausible explanation for our findings is that low-dose alcohol suppresses tremor by enhancing GABA-mediated tonic currents in CGCs by activating α6βδ GABA_A_ receptors. This interpretation is consistent with the finding that alcohol at 10 mM (equivalent to 0.05 g/dl) enhances GABA-mediated tonic currents in slices of CGCs [16]. This level is comparable to blood levels that suppress ET tremor [5,6] and to estimated blood levels found to suppress harmaline tremor. This interpretation is also compatible with high-density EEG evidence that alcohol acts on the cerebellum [8] and with observations that alcohol reduces cerebellar hypermetabolism [9,10].

We also found that the neurosteroid ganaxolone, in doses that do not cause impairment on the straight-wire test, reduces tremor in WT mice, but not in littermates lacking the α6 or δ subunit. Neurosteroids activate α6βδ and α4βδ extra-synaptic GABA_A_ receptors [23] and, like alcohol, are positive allosteric modulators, enhancing GABA-mediated tonic inhibition, but bind to different sites on the receptor [41,42,43]. In addition, using similar methodology, we have previously reported that gaboxadol suppresses harmaline tremor in WT mice at doses that do not cause psychomotor impairment, but fails to suppress tremor in littermates lacking the α6 or δ subunit [44]. In contrast to the positive allosteric action of ethanol and ganaxolone, gaboxadol is a selective agonist of extra-synaptic GABA_A_ receptors [45]. Overall, we have found that three compounds that activate extra-synaptic δ GABA_A_ receptors, but bind to different receptor sites, each suppress harmaline tremor in α6- and δ-dependent fashion.

α6βδ GABA_A_ receptor-mediated reduction of CGC activity would reduce parallel fiber firing, and hence PC simple spikes (SSs). We postulate that the downstream effect is to reduce excessive PC complex spike (CS) synchrony, thereby reducing tremor [19]. A PC CS is a spike burst triggered at the climbing fiber-PC synapse [46]. Within small regions of cerebellar cortex PC CSs are dynamically synchronized by clusters of climbing fiber-projecting IO neurons that are coupled via gap junctions [47]. When PC CSs are more synchronized, their convergent projection to DCN neurons is more effective in producing inhibition [48,49], promoting hyperpolarization-induced rebound bursting [50] that is transmitted to the thalamus. Intra-IO Injection of the GABA_A_ receptor antagonist picrotoxin promotes IO coupling, increases PC CS synchrony, and the amplitude of evoked movement in rats [51], and in some animals elicits tremor in association with increased PC CS synchrony [52]. Two other agents that increase IO coupling, systemic harmaline and intra-olivary serotonin receptor 2a agonists [53,54,55], also increase PC CS synchrony [55,56] and induce tremor [54,57]. *Hotfoot17* mice exhibit ET brain-like features of aberrantly increased terminal climbing fiber innervation of multiple PCs [58]; tremor occurs that depends, as with harmaline tremor, on an intact IO [58,59], intact climbing fiber-PC synapses [53,54,58], and on GABA release from PC axon terminals in DCN [58,60]. In *hotfoot17* mice, increased PC synchrony appears mainly due to aberrant climbing fiber multi-PC innervation [58]. Local field potentials reveal cerebellar oscillations that are coherent with both IO bursting and with tremor in *hotfoot17* mice [58]. Interestingly, the majority of ET patients also display cerebellar oscillations [58], suggesting that increased PC CS synchrony may underlie ET tremor.

The degree of IO coupling, and hence PC CS synchrony, is modulated by afferents to the IO. Intra-IO GABA release inhibits coupling, thereby reducing PC CS synchrony [52,61]. The main afferent source of GABA to IO is the massive GABAergic projection from DCN [62]. These IO-projecting DCN neurons in turn receive GABAergic projections from PCs as their main input and appear to integrate ongoing activity [63,64], such as PC SSs. Because PCs receive climbing fibers from IO cells that receive afferents from DCN neurons to which they project (a tri-synaptic circuit), PC SSs can influence CS synchrony within the same cerebellar cortical region. When picrotoxin is applied to cerebellar cortex in anesthetized rats, local PC SSs increase, and PC CS synchrony also increases via the tri-synaptic circuit [65]. Similarly, if CGC hyperactivity underlies cerebellar hypermetabolism that occurs in ET [9,10], the resulting increase in PC SSs could exacerbate tremor by promoting PC CS synchrony which, as discussed above, underlies tremor in the harmaline and *hotfoot17* animal models, and is the probable basis of cerebellar oscillations in ET. Application of the GABA_A_ receptor agonist muscimol to rat cerebellar cortex leads to reduced PC SSs and reduced CS synchrony [65]. In this case, reduced PC SS firing disinhibits DCN neurons so that they release more GABA within IO, reducing coupling and downstream PC CS synchrony. In parallel, Boecker et al. [9] found that low-dose alcohol reduces cerebellar hypermetabolism in ET patients and moreover increases metabolism in the region of the IO, which they interpreted as due to increased DCN axonal firing [9], comparable to muscimol’s tri-synaptic circuit action in rats [65].

A potential limitation is that we did not perform Western blot to confirm reductions of α6 or δ subunits in KO mice. However, such reductions are well-established in such mice identified by genotyping. A limitation was that we did not study the effect of alcohol or ganaxolone on α4 KO mice. As doses causing psychomotor impairment are likely in part due to activation of α4βδ extra-synaptic GABA_A_ receptors present in many brain areas, the study of these drugs in α4 KO mice might have allowed higher doses to be tested that do not cause psychomotor impairment, thereby providing insight into the potential efficacy of α6βδ-selective drugs. For example, in WT mice, gaboxadol at 10 mg/kg causes sedation and impaired rotarod performance, whereas α4 KO mice do not exhibit such impairments although the drug is free to act on α6βδ GABA_A_ receptors in these mice [66]. Similarly, ganaxolone exhibited only moderate efficacy against tremor in WT mice in the present study, but might have exhibited more efficacy in α4 KO mice if they tolerate higher doses.

The GABA hypothesis of ET postulates that a disturbance of GABA function occurs in ET [67]. In the circuit discussed above, synchronous GABA release from PC terminals in DCN is required for tremor expression, whereas GABA receptor activation in IO or CGCs can suppress tremor by reducing PC synchrony. This concept offers a framework for considering how dysfunction of GABA transmission may contribute to tremor. However, the present finding that activation of α6βδ GABA_A_ receptors suppresses tremor in the harmaline model does not necessarily implicate dysfunction of these receptors in ET. Cerebellar cortical hypermetabolism in ET [9] may be due, for example, to heightened afferent excitatory drive rather than intrinsic GABA receptor dysfunction.

The tri-synaptic pathway concept offers a mechanism how positive allosteric modulators or agonists of α6βδ extra-synaptic GABA_A_ receptors on CGCs, such as low-dose alcohol, ganaxolone, and gaboxadol, could suppress tremor, via a downstream effect on PC CS synchrony. Since the effect of alcohol in ET is to normalize cerebellar metabolism [9], a selective α6βδ receptor modulator may be well-tolerated. However, none of these compounds are selective for α6βδ receptors; to our knowledge there are currently none. The concurrent activation of α4βδ GABA receptors, for example, is problematic, as this may be associated with undesirable effects [44]. Our results suggest that the α6βδ receptor is a promising target for drug development. As CGCs also strongly express α6βγ2 GABA_A_ receptors, these may also constitute a viable therapeutic target.

## Data accessibility statement

Data are available from the corresponding author upon reasonable request.
